# Metaphoric language in the differential diagnosis of epilepsy and psychogenic non-epileptic seizures: Time to move forward

**DOI:** 10.1016/j.ebr.2023.100639

**Published:** 2023-12-21

**Authors:** Lina Urh, Daniele Piscitelli, Massimiliano Beghi, Silvia Diotti, Giuseppe Erba, Adriana Magaudda, Mikhail Zinchuk, Alla Guekht, Cesare Maria Cornaggia

**Affiliations:** a School of Medicine and Surgery, University of Milano Bicocca, Milan, Italy; b Department of Kinesiology, University of Connecticut, Storrs, CT, USA; c Department of Mental Health, AUSL Romagna, Cesena, Italy; d Department of Neurology, University of Rochester, USA; e Epilepsy Centre, Neurological Clinic, University of Messina, Italy; f Moscow Research and Clinical Center for Neuropsychiatry of the Healthcare Department of Moscow, Moscow, Russia

**Keywords:** Metaphors, Psychogenic seizures, Nonepileptic seizures, Epileptic seizures, Conversation analysis

## Abstract

Conversation analysis (CA) to identify metaphoric language (ML) has been proposed as a tool for the differential diagnosis of epileptic (ES) and psychogenic nonepileptic seizures (PNES). However, the clinical relevance of metaphoric conceptualizations is not clearly defined. The current study aims to investigate the ML utilized by individuals with ES and PNES in a pulled multi-country sample. Two blinded researchers examined the transcripts and videos of 54 interviews of individuals (n = 29, Italy; n = 11, USA; n = 14, Russia) with ES and PNES, identifying the patient-seizure relationship representative of the patient's internal experience. The diagnoses were based on video-EEG. Metaphors were classified as “Space/place”, “External force”, “Voluntary action”, and “Other”. A total of 175 metaphors were identified. No differences between individuals with ES and PNES were found in metaphoric occurrence (χ^2^ (1, N = 54) = 0.07; p = 0.74). No differences were identified when comparing the types of metaphors utilized by participants with ES and those with PNES. Patients with PNES and ES did not demonstrate differences in terms of occurrence and categories in ML. Therefore, researchers and clinicians should carefully consider the use of metaphor conceptualizations for diagnostic purposes.

## Highlights

• Conversational analysis may distinguish between ES and PNES.
• No differences were found in metaphoric language between ES and PNES.
• Metaphor conceptualizations in ES and PNES diagnosis should be used with caution.

## 1 Introduction

Several studies have demonstrated the clinical application of conversation analysis (CA) for the differential diagnosis of epileptic (ES) and psychogenic (or paroxysmal) nonepileptic seizures (PNES) [1], [2], [3], [4], [5], [6], [7], [8], [9], [10], [11], [12], otherwise called “functional seizures” [13] or “dissociative seizures” [14]. CA is a promising alternative to the current gold standard of video-electroencephalography (v-EEG), developed through the examination of semi-structured interviews. Notably, the same key linguistic and semantic characteristics have been identified to differ between ES/PNES in German [1], [2], [3], English [4], [5], [6], Italian [6], [7], [8], [9], French [10], Russian [11] and Chinese [12].

Recently, five semantic characteristics, namely: I) focus of the narrative (seizure vs. environment); II) role of the body (active vs. passive); III) changes in speech; IV) presence/absence of gap; and V) expressive intent (aiming to explain vs. impress), were summarized into a scoring table [6], [11], [15], reviewed in 16], which simplified the process of Conversation Analysis (CA). However, this summary excludes one of the fundamental aspects of CA previously utilized: metaphoric language (ML) [1], [2], [3], [4], [5], [7], [8], [9].

Metaphoric language has been defined by Lakoff & Johnson as the “understanding and experiencing one kind of thing in terms of another” [17]. In other words, ML combines psychologic and linguistic processes, transposing one reality with another based on a perceived similarity [18]. Research of ML in health communication has traditionally concentrated on idiosyncratic metaphors used by individual patients or practitioners [19]. Many of the studies on this topic [20] use data in which patients explain how they feel *about* being ill, rather than how they feel *during* an acute episode. Finding conventional metaphorical mappings in the latter could be useful, since the actual experience of the symptoms may be far removed from ordinary everyday thinking and reasoning.

To the best of our knowledge, Plug et al. [21] is the only study to focus specifically on the aspect of ML in CA, finding a significant difference between participants with ES and those with PNES in a sample of 21 individuals. While their findings are promising, the generalizability of the study is limited by the relatively small sample size and examination of only patients speaking English.

The current study aims to examine the ML utilized by participants with ES and PNES in a pulled multi-country sample study, and to investigate whether ML can enhance the differential diagnosis of ES/PNES in a clinical setting.

## 2 Materials and methods

### 2.1 Participants and interviews

The sample population consisted of patients enrolled in the University of Milano Bicocca and the Epilepsy Center of the University of Messina (Italy), the Moscow Research and Clinical Center for Neuropsychiatry (Russia), and the Epilepsy Monitoring Unit of the University of Rochester (USA) for the purpose of previous CA studies [6], [7], [8], [11]. The diagnosis of PNES was obtained with v-EEG monitoring of the events. Witnesses of the seizures were asked to verify whether the captured seizures were representative of the habitual events experienced by the patients. In case of an unclear diagnosis or the coexistence of ES and PNES, the patients were excluded.

Inclusion criteria included a normal intelligence quotient and fluency in the language of communication. Age was not utilized as an inclusion parameter, since a previous CA study demonstrated the presence of the same semantical differences in the pediatric and adolescent settings [8]. All participants underwent a semi-structured video interview, subsequently analyzed for the use of metaphoric language. To establish a differential diagnosis between ES/PNES, v-EEG was utilized in all cases. Witnesses of the seizures were asked to verify that the captured seizures were representative of the habitual events experienced by the patients. Patients with an unclear diagnosis or with a coexistence of ES and PNES were excluded.

The following five predetermined questions were administered to all participants: I. What do you expect from today's meeting? II. Could you tell me about your very first seizure? III. Could you tell me about your very last seizure? IV. Could you tell me about your worst seizure? V. What do you like to do in your spare time? The details of the interview are described elsewhere [4], [5], [6], [7]. All participants and interviewers were fluent in the language of conversation. ES/PNES diagnoses were based on v-EEG. Informed consent was obtained from all participants (or their legal guardians). The study complies with the Declaration of Helsinki.

### 2.2 Metaphoric language

The transcripts and videos of the interviews were re-examined by two authors (SD, LU), each fluent in at least two of the interview languages, to identify relevant metaphors. The two authors were instructed by a consulting linguist on the proper identification and categorization of ML. Note that the two authors (SD, LU) were blind to the diagnosis of the participants.

ML was defined as described by Lakoff & Johnson [17]. Researchers identified phrases describing and characterizing the participant's relationship with the seizure. ML related to the physical manifestations and sensations during a seizure was not included in the analysis. For instance, “*like ants crawling on my skin*” was removed from the analysis because it describes a feeling accompanying the seizure, but not the seizure itself. Rather, “*it [the seizure] rips me away*” refers directly to how the subject experiences the seizure. The focus was on metaphors concerning the direct patient-seizure relationship since only those express the patient's internal experience. This distinction is in line with the CA performed in previous studies [7], [8].

Based on the imagery conjured by the identified sentences, the metaphors were divided into four types, similar to the study by Plug et al. [19]: seizure as space/place, seizure as external force, seizure as voluntary action, and seizure as other. The category “seizure as space/place,” was defined by the description of a space that the speaker moves into, out of, or through (e.g., “flew out of“). Instead, the category ”voluntary action“ was defined by phrases that gave agency to the speaker rather than the seizures; the phrase ”*I shut off*“ places the individual as the acting agent. In contrast to the example previously given, ”*it [the seizure] rips me away*“, centers the seizure as the propagating force and thereby belongs to the ”external force“ category.

### 2.3 Statistical analysis

All data were presented as mean and standard deviation (SD) or frequency with percentage unless otherwise indicated. To investigate demographic differences between ES and PNES subjects independent U Mann Whitney and chi-square (χ^2^) statistics were used. Independent t-tests were applied to analyze metaphor type differences in participants with ES and those with PNES. An additional linear mixed-effects regression was performed to analyze the variability between centers, reported as variance (σ^2^) and standard error (SE). Statistical Package for the Social Sciences (SPSS) version 22 was used for all statistical analyses. Significance level was set at p ≤ 0.05.

## 3 Results

The semi-structured video interviews of 54 participants, 24 (44 %) with ES and 30 (56 %) with PNES, were analyzed. More specifically, 29 patients (ES: 11, PNES: 18) came from the Italian centers, 11 patients (ES: 7, PNES: 4) from the American center, and 14 patients (ES: 6, PNES: 8) from the Russian center.

The duration of interviews ranged from 8:13 to 35:40 min with an average of 19:58 min ± 07:02 (mean ± SD).

Participants' average age was 31.8 ± 16.3 years (mean ± SD; range = 8–66 years). Age distribution showed no significant differences between ES (33.6 ± 17.5 years, mean ± SD) and PNES (30.5 ± 15.5 years, mean ± SD), t(48) = 0.65, p = 0.43, 95 % CI [-6.34, 12.43]. Of the total population, 16 (30 %) of the participants were male, while 38 (70 %) were female. When comparing the ES and PNES populations, no significant differences were observed for gender composition (χ^2^ (1, N = 54) = 0.004, p = 0.95); both groups had a higher proportion of females (ES vs PNES, 71 % vs 70 %).

A total of 175 metaphors were identified among the 54 participants. ML was utilized by 67 % (n = 16) of the participants with ES, compared to 63 % (n = 19) of participants with PNES (χ ^2^ (1, N = 54) = 0.07, p = 0.80). The U Mann Whitney test for non-parametric variables showed non-significant differences between the ES and PSES groups (p = 0.74). Similarly, no significant differences were identified when comparing the types of metaphors utilized by participants with ES and those with PNES (See Table 1).

**Table 1:** Utilization of metaphor types in patients with ES and those with PNES.

**Seizure as:**	**ES**						**PNES**						**Statistics**		
	Mean	SD	Median	Min	Max	Range	Mean	SD	Min	Median	Max	Range	df	t	p
***Space/place***	1.08	2.04	0	0	8	8	1.07	1.70	0	0	6	6	52	0.03	0.97
***External force***	1.21	2.09	0	0	7	7	0.80	1.40	0	0	5	5	52	0.86	0.39
***Voluntary action***	0.5	1.47	0	0	7	7	0.40	0.86	0	0	4	4	52	0.31	0.76
***Other***	0.7	1.73	0	0	8	8	0.60	1.10	0	0	4	4	52	0.28	0.78

ES- Epileptic seizure; PNES- Psychogenic nonepileptic seizure.

When dividing metaphor use based on language, the data suggest a greater propensity for Russian-speaking patients to utilize metaphoric language, with 50 % of patients utilizing 3 or more metaphors, compared with 28 % of Italian-speaking patients and 27 % of English-speaking patients (p = 0.02)(See Table 2).

**Table 2:** Metaphor use across languages.

**Language**	**0 metaphors**	**1**–**2 metaphors**	**3 + metaphors**	**P value**
Italian	15 (52 %)	6 (21 %)	8 (28 %)	
English	1 (9 %)	7 (64 %)	3 (27 %)	
Russian	3 (21 %)	4 (29 %)	7 (50 %)	p = 0.0200

Based on the linear mixed-effects regression analysis performed, the center at which the interview was performed did not have a significant impact on the number of metaphors (σ^2^ 0.6, SE 1.5) or metaphor type (space/place: σ^2^ 0.6, SE 0.7; external force: σ^2^ 0, SE 0; voluntary action: σ^2^ 0.3, SE 0.4; other: σ^2^ 0, SE 0).

Inter-rater agreement, based on the analyses of 5 patients, reached 92 %, with a Cohen’s κ of 0.75, indicating substantial inter-rater reliability.

## 4 Discussion

Seizures are complex physiological events that are experienced by patients in terms of sensation and changes of state. In our study, while all patient groups averaged at least one metaphor per interview, many patients did not make use of ML at all and only two-thirds of patients utilized metaphoric language describing their direct relationship with seizures during the interview. Moreover, the difference in metaphor use between patients with ES and those with PNES, both in terms of frequency and content, does not reach a statistical significance.

These findings contrast those of Plug et al. [21], the only reference examining ML utilization in patients with ES and those with PNES. Plug and colleagues found a significant difference in the type of metaphor preferred by the two groups. Patients with PNES preferred language conjuring the imagery of passing through or into the seizure as though it were a space/place. Instead, patients with ES had a greater inclination towards describing their seizures as an enemy or an external force to be defeated, echoing the Greek origin of the word itself: epilambanein (ἐπιλαμβάνω), to be taken ahold of. These distinctions were not found significant in this study.

The methodology of our study is comparable with that of Plug and colleagues. The difference in results is likely due to a reduced number of identified metaphors per patient in this study. The definition of Lakoff & Johnson [17] is broad, which allows for much room for interpretation. Unlike Plug and colleagues, we did not consider all verbs of motion describing seizures as ML, considering them “literal”. Previous work on metaphor in health communication has highlighted the fact that the boundary between the literal and the metaphorical is fuzzy rather than clear-cut [22]. A phrase such as “*the seizure started*” was not included since it was considered a generic expression dictated by the limitations of language (literal), rather than a representation and description of the relationship between patient and seizure (metaphoric). In contrast, a phrase such as “it [the seizure] rips me away,” creates a clear image of the dynamic present between patient-seizure. Verbs of motion were included only when there was a clear construction of metaphor and the seizure as the agent of motion. Plug and colleagues [21] linguistically justify their decision, however in our opinion, their broad definition of ML limits its feasibility in a practical clinical setting.

It is interesting to note the increased tendency of Russian-speaking patients to use metaphoric language. This could be a consequence of language structure and/or cultural differences. As Boris B. Bogoslovsky pointed out in The Russian Review, every language possesses its own strong points: Italian is the language of passion and drama, English a language of direct and simple communication, and Russian a language rich in the expression of nuanced moods and intentions. The cultural history of Russia has, for centuries, placed great emphasis on lyrical contemplation and philosophical speculation [23]. The propensity of Russian to adopt figurative language has also been remarked upon by Thomas Seifrid [24] in his discussion of organic metaphors, where he states that “it [organic metaphors] assumes a central place… it took up this position in Russian thought because it fit in well with more deeply embedded notions about language and selfhood traceable to Russia’s medieval past”. This, in addition to the inherent figurative meaning held by Russian verbs of motion, seems to imply an innate tendency of Russians to utilize metaphoric language during their speech.

Alternatively, the differences in metaphor use between languages may be also due to demographic differences, such as age or education, which have not been accounted for. For instance, pediatric patients are included only in the Italian group, which presents a low use of ML; 52 % of patients do not utilize ML at all. Instead, education status is known only of the Russian patients, with 50 % having completed (or were in the process of completing) a university level degree.

## 5 Limitations

This study has several limitations. Firstly, the SD of each number of metaphors was very high compared to the mean for all categories and we were not able to evaluate if there were consistent confounding factors (demographic or clinical) that may account for the variability (i.e. age, education, site-related differences, length of the illness, psychiatric or somatic comorbidity). Secondly, while the interviews were rigorously structured for consistency and interviewers limited to as few as possible, some differences in patient response may be due to the interviewer and their style. Furthermore, we did not control or evaluate the extent to which patients had felt stigmatized by their illness. Lastly, our study is not a direct replica of the one conducted by Plug et al. [21]. Therefore, the lack of agreement between the two studies may stem from inconsistent results, methodology/definition, or a combination of both.

## 6 Conclusions

In recent years, CA has emerged as a reliable tool for the differential diagnosis between ES and PNES. ML was one of the key elements examined for the differential diagnosis, but the recently developed simplified scoring table has shown promising results even in the absence of ML analysis. According to our results, it seems that the subjective nature of the definition of ML hinders ML analysis (both in terms of frequency and content) from being an effective tool in the differential diagnosis of ES/PNES.

## Declaration of interest

None.

## Ethical statement

We confirm that we have read the Journal’s position on issues involved in ethical publication and affirm that this report is consistent with those guidelines. The manuscript presents secondary data analysis. Informed consent was obtained from all participants (or their legal guardians). The study was performed following the Declaration of Helsinki.

## Data availability statement

The data that support the findings of this study are available from the corresponding author [DP] upon reasonable request.

## Funding statement

This research received no funding.

## CRediT authorship contribution statement

**Lina Urh:** Writing – original draft, Investigation, Formal analysis. **Daniele Piscitelli:** . **Massimiliano Beghi:** Writing – review & editing, Writing – original draft, Methodology, Data curation, Conceptualization. **Silvia Diotti:** Writing – original draft, Investigation, Formal analysis. **Giuseppe Erba:** Writing – review & editing, Supervision, Investigation, Conceptualization. **Adriana Magaudda:** Writing – review & editing, Methodology, Conceptualization. **Mikhail Zinchuk:** Writing – review & editing, Supervision, Investigation, Data curation. **Alla Guekht:** Writing – review & editing, Methodology, Conceptualization. **Cesare Maria Cornaggia:** Writing – review & editing, Supervision, Investigation, Data curation, Conceptualization.

## Declaration of competing interest

The authors declare that they have no known competing financial interests or personal relationships that could have appeared to influence the work reported in this paper.
